# Enhancing the Hydrophobicity of Chitosan Films Through Hierarchical Plant Wax Particles and Dilute Chitosan Solution Coatings

**DOI:** 10.3390/foods14040610

**Published:** 2025-02-12

**Authors:** Chengcheng Gao, Ni Zhang, Ying Chen, Fengwei Xie, Linghan Meng, Xiaozhi Tang

**Affiliations:** 1 College of Food Science and Engineering, Nanjing University of Finance and Economics/Collaborative Innovation Center for Modern Grain Circulation and Safety, Nanjing 210023, China; hellowgao@126.com (C.G.); rainbowzn98@163.com (N.Z.); menglinghan@nufe.edu.cn (L.M.); 2 School of Food Science and Engineering, Yangzhou University, Yangzhou 225127, China; 008265@yzu.edu.cn; 3 Nottingham Ningbo China Beacons of Excellence Research and Innovation Institute, University of Nottingham Ningbo China, Ningbo 315048, China

**Keywords:** hierarchical structure, hydrophobic coating, carnauba wax, chitosan

## Abstract

The strong hydrophilicity of chitosan-based films limits their practical applications. To enhance the hydrophobicity of these films, hierarchical carnauba wax particles were prepared using the Pickering emulsion method and subsequently coated onto the film surfaces. The wax was stabilized with various types and concentrations of TiO_2_. The resulting wax particles exhibited a micro-scale structure, with nano-scale TiO_2_ and micro-scale TiO_2_ aggregates present on the surface. No significant differences in contact angle were observed among these particles. Hydrophilic TiO_2_ demonstrated smaller sliding angles and particle sizes. To improve the mechanical durability and compatibility of the wax particles with the chitosan matrix, the wax particles were mixed with a diluted chitosan solution before coating. As the concentration of wax particles increased and the concentration of chitosan solution decreased, more wax particles became exposed on the surface. This exposure increased the roughness of the coatings, resulting in a significant increase in contact angle and a decrease in sliding angle. A high concentration of chitosan provided greater protection to wax particles during mechanical durability tests. Additionally, the residue rate of liquid foods on the coating films significantly decreased. This study demonstrates that the Pickering emulsion method is an effective approach for preparing hierarchical wax particles, and that mixing these particles with a polymer similar to the matrix can effectively improve mechanical durability.

## 1. Introduction

The advent of petroleum-based materials has provided immense convenience in daily life; however, the rising consumption of petroleum has led to resource depletion and significant environment impacts. Consequently, the urgent development of renewable, biodegradable materials is essential. Currently, natural polymers, including proteins, polysaccharides, lipids, and other natural molecules and their derivatives, are gaining considerable interest as alternatives to petroleum-based materials in food packaging, biomedical applications, and environmental solutions [1].

Polysaccharides, in particular, offer advantages such as low cost, abundant availability, and relative stability, making them highly promising for the creation of renewable and biodegradable materials [2]. However, the numerous hydroxyl groups present in the polysaccharide chain contribute to their strong hydrophilicity [3]. This property can compromise the integrity of the materials upon exposure to water, negatively affecting their performance. Therefore, enhancing the hydrophobicity of polysaccharide-based materials is crucial [4]. Common methods for achieving the hydrophobic modification of polysaccharide-based materials include blending, chemical modification, multilayer films construction, and hydrophobic coating techniques [5].

Hydrophobic coating involves applying a layer of hydrophobic material to the surface of a substrate. The microstructure of the material’s surface plays a crucial role in determining its hydrophobicity. Nature provides inspiration for this phenomenon, exemplified by lotus leaves, which feature numerous micro- and nano-scale protrusions coated with wax. These structures are primarily responsible for the superhydrophobicity and self-cleaning properties of lotus leaves [6,7].

Various methods exist for creating micro- and nanostructures on substrate surfaces, such as chemical bath deposition, surface etching, chemical vapor deposition, and electrostatic layer-by-layer assembly [8,9]. Recently, our research group pioneered the use of the Pickering emulsion method to prepare plant wax particles. These wax particles are spherical and exhibit a hierarchical structure, with nano-scale and micro-scale TiO_2_ aggregates present on their surfaces. The thermal properties of these wax particles are comparable to those of the original wax, and they also demonstrate antibacterial activity due to the incorporation of TiO_2_. When these wax particles are coated onto chitosan films, they significantly enhance the hydrophilicity of the films [10]. While it is well-established that particle characteristics are vital for the stabilization and structure of Pickering emulsions, it is not clear how the specific effects of TiO_2_ type and concentration affect the structure of plant wax particles produced via this method.

Despite the availability of numerous effective methods for preparing hydrophobic surfaces, many of these surfaces lack mechanical durability and are prone to wear during daily use [11]. To enhance the wear resistance of hydrophobic coatings, common strategies include strengthening the bond between the surface structure and the substrate, developing self-healing hydrophobic materials, utilizing sacrificial hydrophobic materials, and creating multilayer films [12]. However, these approaches may introduce new substances, involve complex processes, and/or result in nonuniformity interfaces [13,14,15]. To address this issue with a simple method, we hypothesize that mixing wax particles with a hierarchical structure with a polysaccharide that matches the matrix can improve the mechanical durability of hydrophobic coatings. In this design, the polysaccharide not only protects the particles from wear, but also enhances compatibility between the wax particles and the matrix by leveraging the excellent interface compatibility of the same material [16,17].

The study aims to improve the hydrophobicity of polysaccharide-based materials and enhance the durability of the hydrophobic covering. This was accomplished by synthesizing hierarchically structured wax particles via a Pickering emulsion method. The effects of different types and concentrations of TiO_2_ on the structure of the wax particles were studied. To further enhance the mechanical durability of the wax particles, they were mixed with dilute chitosan and subsequently coated onto the surface of chitosan films. This study also examined how variations in the concentration of wax particles and chitosan influence the structure and hydrophobicity of chitosan films.

## 2. Materials and Methods

### 2.1. Materials

Carnauba wax (melting point > 83 °C, food grade) was obtained from Shanghai Aladdin Reagent Co., Ltd. (Shanghai, China). Different types of titanium dioxide (99.8%, hydrophilic Anatase, 5–10 nm; 99.8%, hydrophilic–lipophilic Anatase, 5–10 nm; and 99.8%, hydrophilic Anatase, 60 nm) were sourced from Shanghai McLean Biochemical Technology Co., Ltd. (Shanghai, China). Chitosan (85% deacetylation, *M*_w_ = 4.0–5.0 × 10^5^ g mol^−1^) was purchased from Jinan Haidebe Marine Biological Engineering Co., Ltd. (Jinan, China). Polydimethylsiloxane (PDMS, Dow Corning 184) was acquired from Dow Silicones (Midland, MI, USA). Yogurt and honey were purchased from local supermarkets in Jiangsu, China. Glacial acetic acid, hydrochloric acid, and sodium hydroxide were obtained from Sinopharm Group Chemical Reagent Co., Ltd. (Shanghai, China).

### 2.2. Preparation of Hierarchical Wax Particles

Different types of TiO_2_ were dispersed in water with a pH of 5 at certain concentrations (0.7%, 1%, 1.3%, *w*/*v*). These dispersions were treated using an ultrasonic cleaner set at 150 W for 20 min. A specified volume of TiO_2_ dispersion (60 mL) was then placed in a water bath at 90 °C, to which 1 g of carnauba wax was added. The mixture was stirred for 30 min until the carnauba wax was completely melted. High-speed dispersion was performed using an IKA T18 mixer (IKA, Staufen, German) at 12,000 r/min and 90 °C for 5 min. Following this, the mixture was allowed to cool at room temperature for 30 min. The wax particles that settled at the bottom were then extracted and placed in a 50% relative humidity (RH) environment to facilitate water evaporation.

### 2.3. Preparation of Coating Films

A chitosan solution with a concentration of 1.5% (*w*/*v*) was prepared by dissolving a certain amount of chitosan in a 1% (*v*/*v*) acetic acid solution. To create the film-forming solution, an amount of glycerol (20% *w*/*w* glycerol relative to the total solid) was added to the chitosan solution. The chitosan film-forming solution was then diluted to 0.1%, 0.01%, and 0.001%.

**Figure 1:**
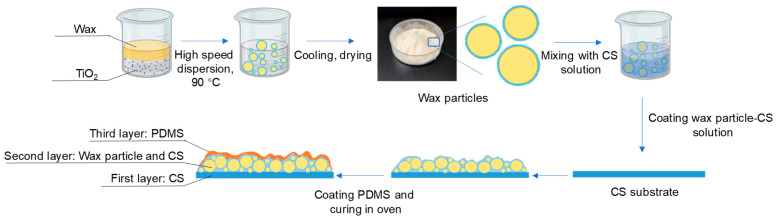
Preparation of wax particles and coating films.

The preparation of coating films is shown in Figure 1.

Preparation of first layer: three grams of the 1.5% (*w*/*v*) chitosan film-forming solution were added to a round petri dish with a diameter of 3.5 cm and left at room temperature to evaporate the water until the solution no longer flowed.

Preparation of second layer: Different weights of wax particles were dispersed in the 0.01% chitosan film-forming solution to create wax particle–chitosan solutions with concentrations of 0%, 7%, 9%, 11%, 13%, and 15% (*w*/*v*). Additionally, a specified amount of wax particles (13% *w*/*v*) was dispersed in chitosan film-forming solutions at different concentrations (0.1%, 0.01%, 0.001%, and 0%, *w*/*v*). One milliliter of each solution was absorbed into the petri dish containing the first layer, which was then dried at room temperature to form a bilayer film.

Preparation of third layer: A PDMS mixture (with a ratio of PDMS precursor to curing agent of 10:1, *w*/*w*) was placed in anhydrous ethanol and subjected to ultrasonic treatment at 150 W for 10 min to achieve a uniform dispersion solution (4% *w*/*v*). The completely dried bilayer film was soaked in the PDMS dispersion solution for 2 min, followed by a 30 s wash in anhydrous ethanol to remove excess PDMS. The anhydrous ethanol was allowed to evaporate at room temperature, and the films were then cured in an oven at 45 °C for 6 h to obtain the final coating films.

The films were designated as follows: CS 4% PDMS, 7% particles 0.01% CS, 9% particles 0.01% CS, 11% particles 0.01% CS, 13% particles 0.01% CS, 15% particles 0.01% CS, 13% particles 0.1% CS, 13% particles 0.001% CS, 13% particles, 0% CS. The designation CS 4% PDMS indicates that no wax particles were added to the film, while 7% particles 0.01% CS signifies that the second layer contained 7% particles and 0.01% CS.

### 2.4. Characterization

#### 2.4.1. Scanning Electron Microscopy (SEM) and Energy-Dispersive X-Ray (EDX) Spectroscopy

The morphology of wax particles and the surface of coating films was analyzed using a scanning electron microscope (Regulus 8100, Hitachi, Tokyo, Japan) at an accelerating voltage of 15 kV. The elemental analysis of the coating films’ surfaces was conducted using energy-dispersive X-ray spectroscopy (Ultim Max 65, Oxford, UK).

#### 2.4.2. Three-Dimensional Laser Microscopy

The three-dimensional imaging and height distribution of wax particles on the surface of coating films were analyzed using a three-dimensional laser microscope (VK-150K, KEYENCE, Osaka, Japan) with an objective lens magnification of 20×.

#### 2.4.3. Water Contact Angle

The contact angle of wax particles and coating films was measured using an optical contact angle measuring instrument (SDC-100S, Dongguan, China). The method was adapted from the report by Zhang et al. [10]. A volume of 2 µL of distilled water was deposited on the surface of the samples at room temperature.

#### 2.4.4. Sliding Angle

Sliding angle was determined based on the methodology described by Zhang et al. [10].

#### 2.4.5. Fourier-Transform Infrared Spectroscopy (FT-IR)

The infrared spectra of the coating films were obtained using a Fourier-infrared spectrometer (Tensor 27, Bruker, Karlsruhe, Germany). Sample preparation and testing methods followed the procedure outlined in the report by Zhang et al. [10].

#### 2.4.6. Mechanical Durability Test

To assess mechanical durability, rough 800-mesh sandpaper was placed on top of the coating films. A 500 g weight was positioned on the sandpaper, which was then pulled back and forth for a distance of 10 cm to simulate friction. Following this, contact angle measurements and SEM analysis were conducted.

#### 2.4.7. Liquid Food Residue Test

A certain weight (*W*_1_) of honey or yogurt was applied to the surface of the coating films, which were then tilted vertically. If the food did not drip in 30 s, the weight of the liquid food (*W*_2_) was recorded. The residual rate of the liquid food on the films was calculated using the following formula:

Residual rate (%) = *W*_2_/*W*_1_ × 100

### 2.5. Statistical Analysis

Mapping was performed using Origin 8.5 software. The significance of the results was assessed using Duncan’s method (*p* < 0.05) with SPSS Statistics software version 24. Different letters indicate significant differences between samples (*p* < 0.05). Each test was conducted in triplicate unless otherwise specified.

## 3. Results and Discussion

### 3.1. Morphology of Carnauba Wax Particles

**Figure 2:**
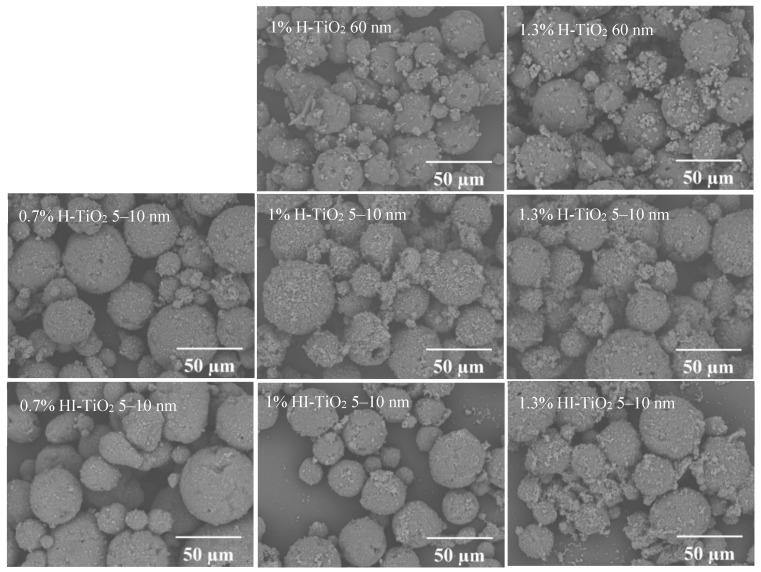
SEM images of different wax particle samples.

The morphology of the different wax particle samples is shown in Figure 2. SEM images reveal that the carnauba wax particles, produced via the Pickering emulsion method, were micron-sized (0–60 µm) and exhibited hierarchical spherical structures. These particles featured a uniform distribution of nanometer-scale and micrometer-scale TiO_2_ aggregates on their surfaces.

The type and size of TiO_2_ did not significantly affect the morphology of the wax particles. Notably, the micro- and nano-structures on the surfaces of wax particles stabilized by 5–10 nm TiO_2_ were smaller than those stabilized by 60 nm TiO_2_. Additionally, the number of micro- and nano-structures on the surfaces of wax particles stabilized by hydrophilic TiO_2_ was greater than those stabilized by hydrophilic–lipophilic TiO_2_, suggesting that hydrophilic TiO_2_ has a stronger tendency to absorb at the oil–water interface. Moreover, the reduced presence of hydrophilic–lipophilic TiO_2_ at the interface resulted in decreased stability of the wax particles, leading to some disaggregation. As the concentration of TiO_2_ increased, the size of TiO_2_ aggregates also grew. For 5–10 nm TiO_2_, a concentration of 0.7% was sufficient to stabilize the wax; however, further increases in TiO_2_ concentration resulted in excess free TiO_2_ becoming visible either in the field of view or on the surface of the wax particles. In contrast, for 60 nm TiO_2_, a concentration of 1% was required for stabilization, while wax particles could not be formed at a concentration of 0.7%.

### 3.2. Wettability of Carnauba Wax Particles

**Figure 3:**
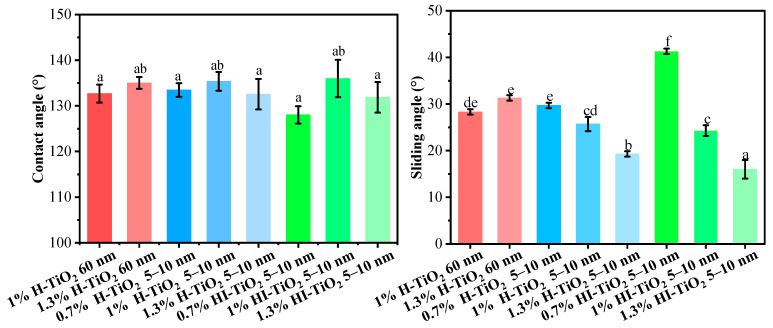
Contact angle and sliding angle values for different wax particle samples. Different lowercase letters indicate significant differences among samples.

The contact angle and sliding angle of the different wax particle samples are presented in Figure 3. As shown, the contact angle of all wax particle samples was approximately 130°, with no significant differences observed based on the type, particle size, and concentration of TiO_2_. This indicates that the hydrophobicity of these particles was consistent across the samples.

For the average contact angle of wax particles stabilized by 5–10 nm hydrophilic and hydrophilic–lipophilic TiO_2_, those stabilized by 1% TiO_2_ exhibited a higher contact angle compared to those stabilized by 0.7% and 1.3% TiO_2_. A moderate concentration of TiO_2_ on the surface of the wax increased the roughness of the particles, thereby increasing the contact angle. Conversely, both lower and higher concentrations of TiO_2_ resulted in the formation of hierarchical structures with either smaller or larger discrepancies among different scales (as observed in SEM), which did not favor the support of water droplets.

The sliding angle represents the angle at which a water droplet begins to roll off the surface of a material. A smaller sliding angle indicates that water droplets can roll off more easily, suggesting better self-cleaning properties. For 60 nm hydrophilic TiO_2_, the content had a negligible effect on the sliding angle. However, for 5–10 nm hydrophilic TiO_2_ and hydrophilic–lipophilic TiO_2_, the sliding angle decreased as the TiO_2_ content increased. The presence of additional TiO_2_ provided more support points for water droplets, facilitating their roll-off. In addition, 5–10 nm hydrophilic TiO_2_ demonstrated lower sliding angles compared to hydrophilic–lipophilic TiO_2_.

From an economic standpoint, and considering both the particle size and wetting properties of the wax particles, 0.7% 5–10 nm hydrophilic TiO_2_-stabilized wax particles were finally selected for the fabrication of coating films.

### 3.3. Morphology and Elemental Analysis of Coating Films

**Figure 4:**
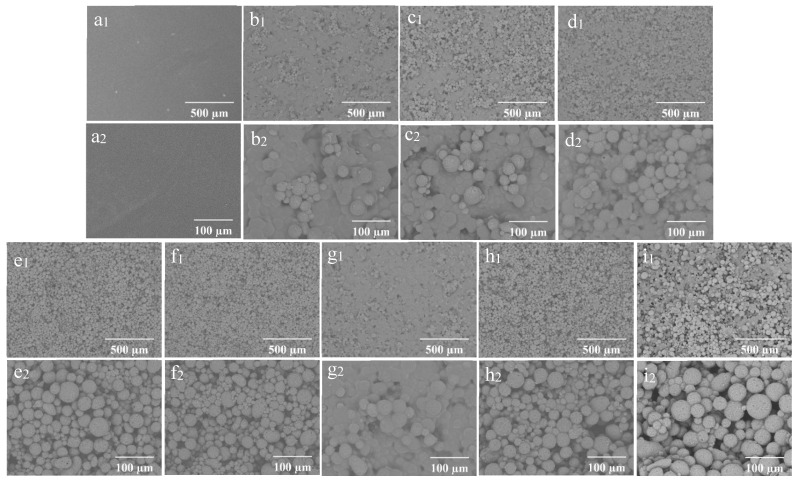
SEM of the coating films (1) and enlarged image (2); (**a**) CS 4%PDMS; (**b**) 7% particles 0.01% CS; (**c**) 9% particles 0.01% CS; (**d**) 11% particles 0.01% CS; (**e**) 13% particles 0.01% CS; (**f**) 15% particles 0.01% CS; (**g**) 13% particles 0.1% CS; (**h**) 13% particles 0.001% CS; (**i**) 0% CS 13% particles.

The SEM morphology of the coating films prepared with different concentrations of wax particles and CS is shown in Figure 4. The coating film’ surface without particles appeared smooth (Figure 4(a1,a2)), while the presence of particles was distinctly observable on the surfaces of the coating films containing them. As a consistent CS concentration with a low concentration of wax particles, most of the particles were embedded within the CS film (Figure 4(b1)). The enlarged image reveals that the micro-/nano-structure of the particles was not discernable (Figure 4(b2)). As the concentration of wax particles increased, a greater number of particles became visible on the surface, gradually exposing the TiO_2_ structure of the wax particles. The wax particles were evenly distributed across the film’s surface (Figure 4(e1)), and the micro-/nano-structure of the wax particles was clearly identifiable (Figure 4(e2)). Further increases in particle concentration did not significantly alter the morphology. When the concentration of particles remained constant, the particles on the surface became submerged in the CS solution as the concentration of CS increased (Figure 4(g1,g2)). Conversely, as the concentration of CS decreased, the morphology of the particles became increasingly apparent. At a CS concentration of 0.01%, the complete morphology of the particles was fully visible (Figure 4(e1,e2)).

**Figure 5:**
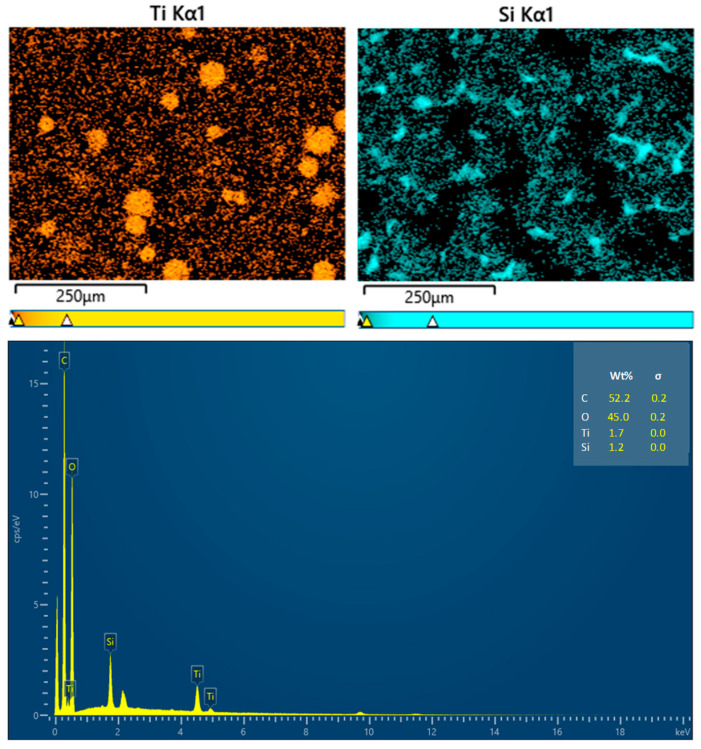
EDX analysis results for the coating films.

The elemental analysis of the coating films is presented in Figure 5. The presence of the Ti element is ascribed to the TiO_2_ coating on the surfaces of the wax particles, while the Si element originates from PDMS. The signatures of C and O may stem from carnauba wax, TiO_2_, PDMS, and CS. The distribution of Ti was primarily observed in circular patterns, confirming that TiO_2_ was indeed present on the wax particles’ surfaces. In contrast, the Si element was generally evenly distributed across the film’s surface, although some areas remained black, indicating that PDMS partially covered the wax particles.

**Figure 6:**
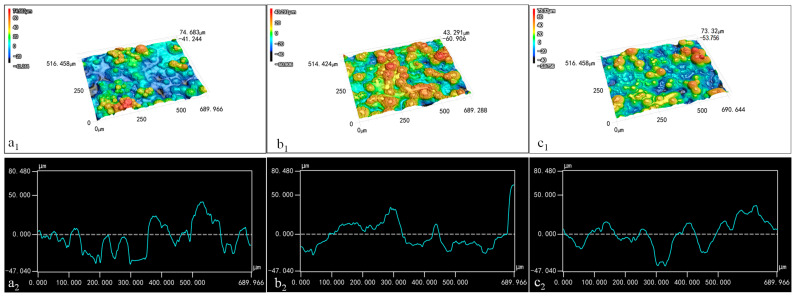
Three-dimensional images (1) and contour curves (2) of the coating films: (**a**) 7% particles 0.01% CS; (**b**) 13% particles 0.01% CS; (**c**) 13% particles 0.1% CS.

The distribution of wax particles on the films’ surface is depicted in Figure 6. Due to the microscope’s low resolution, TiO_2_ and its aggregates on the surfaces of the spherical particles could not be clearly distinguishable. In the three-dimensional image, blue represents the recessed areas, while red indicates protruding structures; darker color signifies greater distance from the plane. When the CS concentration was maintained at 0.01% and the wax particle concentration was 7%, the three-dimensional image displayed more blue light areas and fewer red protrusions (Figure 6(a1)). This suggests that the wax particles were largely submerged within the CS film due to the low concentration of wax particles. Conversely, when the concentration of wax particles increased to 13%, numerous red protruding structures emerged in the three-dimensional image (Figure 6(b1)), indicating that more wax particles were exposed on the surface of the CS film as a result of the higher wax particle concentration.

With the wax particle concentration held at 13%, the three-dimensional image for the coating film containing 0.1% CS solution predominantly showed blue-yellow tones (Figure 6(c1)), indicating that the increased CS concentration significantly covered the particles, thereby reducing the protrusion structures on the film’s surface.

The profile diagram illustrates the shape of the coating film. In Figure 6(a2,c2), the side contour of the film exhibited slight fluctuations, indicating that the combination of low wax particle concentration and high CS concentration resulted in the covering of wax particles. Moreover, the smooth curves in these images suggest that the micro-/nano-structures on the wax particles’ surfaces had diminished, likely due to capillary action filling the spaces between these structures. In Figure 6(b2), the film profile shows significant fluctuations, indicating that the wax particles were fully observable when the wax particle concentration was 13% and the CS concentration was 0.01%. In addition, the rougher curve indicates that TiO_2_ and its aggregates on the surfaces of the wax particles were exposed to a notable extent.

### 3.4. FTIR Spectroscopy

**Figure 7:**
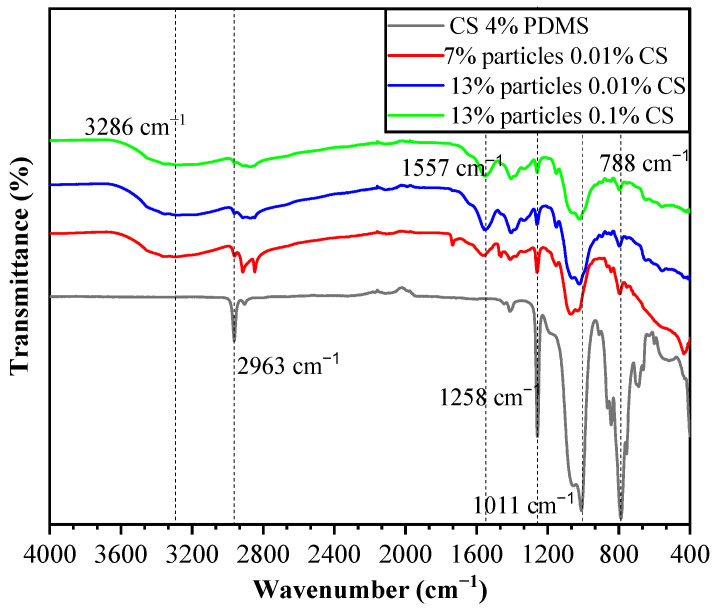
FTIR spectra of the coating films.

The FTIR spectra of the coating films are presented in Figure 7. In the CS 4% PDMS sample, the peaks at 2963 cm^−1^, 1258 cm^−1^, and 788 cm^−1^ were characteristic of the Si-CH_3_ group, while the peak at 1011 cm^−1^ was attributed to Si-O-Si (1000–1100 cm^−1^) [18]. Notably, no characteristic peaks of CS were detected in the CS 4% PDMS sample, indicating that the surface of CS was completely covered by PDMS. The FTIR spectra of the coating films also display a broad absorption peak at 3286 cm^−1^, which is associated with the tensile vibrations of intermolecular and intramolecular O-H, -CH_2_OH, -NH_2_, and -NH secondary amides [19]. Additionally, the peak at 1557 cm^−1^ corresponds to the bending vibrations of N-H [20]. These peaks are mainly characteristic of CS, and the observed decrease in the intensity of PDMS peaks indicates that the addition of particles increased the coating films’ surface area,, preventing the 4% PDMS sample from fully covering the surface of the second layer. In addition, the simultaneous appearance of PDMS and CS peaks, along with the absence of characteristic peaks from wax particles, confirms that the wax particles were completely encapsulated within the CS film. The lack of new peaks in the coating films suggests that no chemical reactions occurred among the components.

### 3.5. Wettability of Coating Films

**Figure 8:**
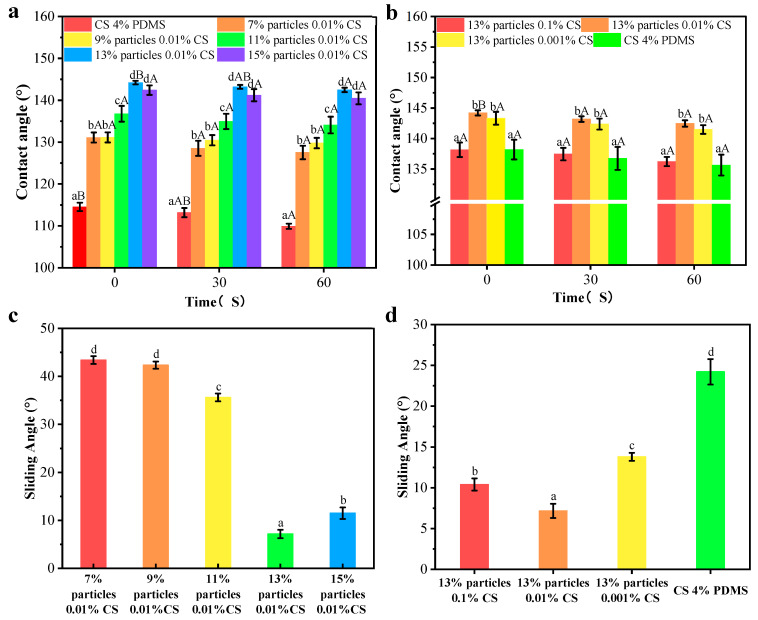
Contact angles and sliding angles of the coating films with different particle concentrations (**a**,**c**) and CS concentrations (**b**,**d**). Different lowercase letters indicate significant differences among samples. Different uppercase letters indicate significant differences among different time points.

The contact angle results are presented in Figure 8a,b. The CS 4% PDMS film exhibited its initial contact angle of 114.54°, which significantly decreased over time, indicating that the incorporation of PDMS enhanced the water resistance of the CS film. As shown in Figure 8a, the addition of wax particles markedly improved the initial contact angle, with the contact angle increasing as the concentration of wax particles rose. The spherical protrusions on the coating films’ surface also increased with higher concentrations of wax particles, which led to a reduction in the surface area where water droplets made contact with the film [21]. When the concentration of wax particle reached 13%, the contact angle peaked at 144.20°. However, further increases in wax particle concentration led to a decrease in contact angle, likely because excess wax particles could not be fully covered by the PDMS (as indicated by FTIR analysis).

In Figure 8b, when the concentration of the CS solution was 0.1%, the coating film exhibited its minimum contact angle of 138.16°. This reduction occurred because the morphology of the wax particles was largely covered by the CS film, diminishing the number of spherical protrusions on the coating film’s surface. As the concentration of CS decreased, more wax particles became exposed, increasing the number of protrusions. However, at 0% CS concentration, the contact angle significantly decreased, possibly due to the complete exposure of the wax particle morphology and a reduced distribution of PDMS per unit area, leading to a lower contact angle. In addition, compared to CS 4% PDMS, the coating films presented no significant change in contact angle indicating improved water resistance. The presence of air trapped in spaces between the wax particles created an air cushion, making it difficult for water droplets to penetrate [22].

The sliding angles of the samples are indicated Figure 8c,d. The CS 4% PDMS film was not included in Figure 8 because it easily absorbed water, preventing water droplets from rolling even when the film was tilted to 90°. In contrast, the sliding angle of all other coating films significantly decreased, demonstrating that the addition of wax particles enhanced self-cleaning performance [23]. As the concentration of wax particles increased, the sliding angle decreased (Figure 8c). At a wax particle concentration of 13%, the sliding angle reached its minimum at 7.17°, attributed to the increased surface roughness from the high concentration of wax particles. Similarly, the sliding angle decreased with lower CS concentrations (Figure 8b), promoting the exposure of wax particles and enhancing surface roughness. Both the increased concentration of wax particles and the decreased concentration of CS effectively decreased the surface interaction between water droplets and the coating films, facilitating water rolling off the surface [24].

However, the sliding angle significantly increased with further increases in wax particle concentration and decreases in CS concentration (Figure 8c,d). Hydrophobicity resulted from the synergistic effect of the microstructure and low surface energy of the surface [25]. The high concentration of wax particles combined with low CS concentration increased the total surface area while decreasing the PDMS content per unit area, resulting in an increased sliding angle. These findings align with the FTIR analysis.

### 3.6. Abrasion Performance of Coating Films

**Figure 9:**
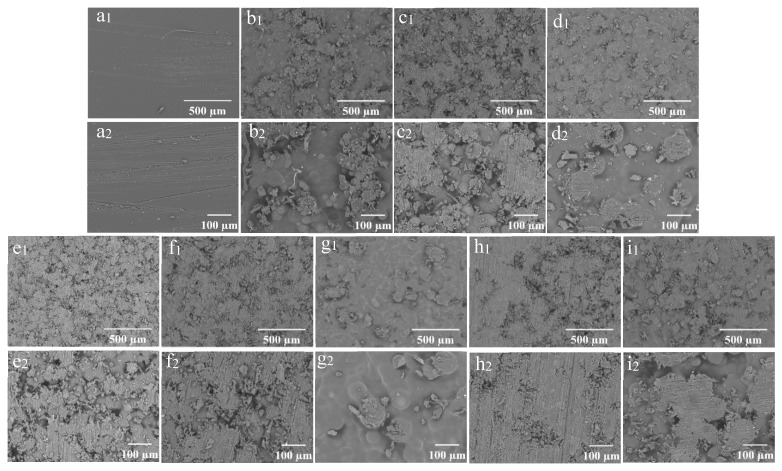
SEM images of the coating films after friction (1) and their enlarged images (2): (**a**) CS 4% PDMS; (**b**) 7% particles 0.01% CS; (**c**) 9% particles 0.01% CS; (**d**) 11% particles 0.01% CS; (**e**) 13% particles 0.01% CS; (**f**) 15% particles 0.01% CS; (**g**) 13% particles 0.1% CS; (**h**) 13% particles 0.001% CS; (**i**) 13% particles 0% CS.

The SEM images of the samples following sandpaper friction are presented in Figure 9. Each film exhibited different degrees of wear on its surface. Notably, scratches were clearly visible on the surface of the CS 4% PDMS film, indicating that the sandpaper friction inflicted significant damage to the CS film. While some spherical protrusions on the coating surface were damaged, others remained intact, indicating that the structure of the films provided a degree of protection for the wax particles.

For the coating films with a constant CS concentration, an increase in the concentration of wax particles resulted in a high number of both damaged and protected particles on the surface (Figure 9(b_2_–e_2_)). However, further increases in wax particle concentration led to a thinning of the CS film over the wax particles, resulting in a greater number of worn wax particles and a decrease in the number of protected particles (Figure 9(f_2_)).

Conversely, for the coating films with a constant wax particle concentration, higher CS concentrations allowed for more wax particles to be incorporated into the film, resulting in fewer worn particles on the surface. As the CS concentration decreased, the wax particles became increasingly exposed, leading to a rise in the number of worn particles. Eventually, at very low CS concentrations, the wax particles were completely worn away, leaving no intact particles. The protective CS film on the surface of the wax particles was thin, making them more susceptible to wear.

**Figure 10:**
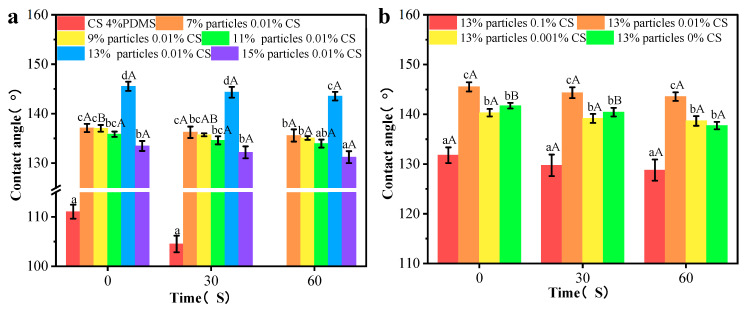
Contact angles of the coating film with different particle concentrations (**a**) and CS concentrations (**b**) after friction. Different lowercase letters indicate significant differences among samples. Different uppercase letters indicate significant differences among different time points.

The contact angle measurements of the coating films are illustrated in Figure 10. After friction, the CS 4% PDMS film displayed an initial contact angle at 111.04°. However, after 60 s, the film swelled, making it difficult to obtain an accurate contact angle. This swelling was attributed to scratches on the surface of the CS 4% PDMS sample, which compromised the integrity of PDMS and hindered its ability to fully protect the CS film.

When different concentrations of wax particles were introduced, the contact angles after wear increased slightly by about 3°. This increase can be attributed to the remaining spherical protruding structures and the newly formed microstructures on the coating surface, as observed in the SEM images. As the concentration of wax particles increased, the contact angle also rose, aligning with the trends observed in the original films.

For the coating films with different CS concentrations, both low and high concentrations of the CS solution significantly decreased the contact angle, in agreement with the SEM results. This reduction was mainly due to the diminished spherical protrusion structures on the surface following friction. Compared to the CS 4% PDMS film the other coating films showed minimal change in contact angle over time. Given this stability, likely, the worn surface structure of the coating films did not expose the CS matrix, thereby preserving the water resistance of the films.

### 3.7. Residue Rate of Liquid Foods on the Coating Films

**Figure 11:**
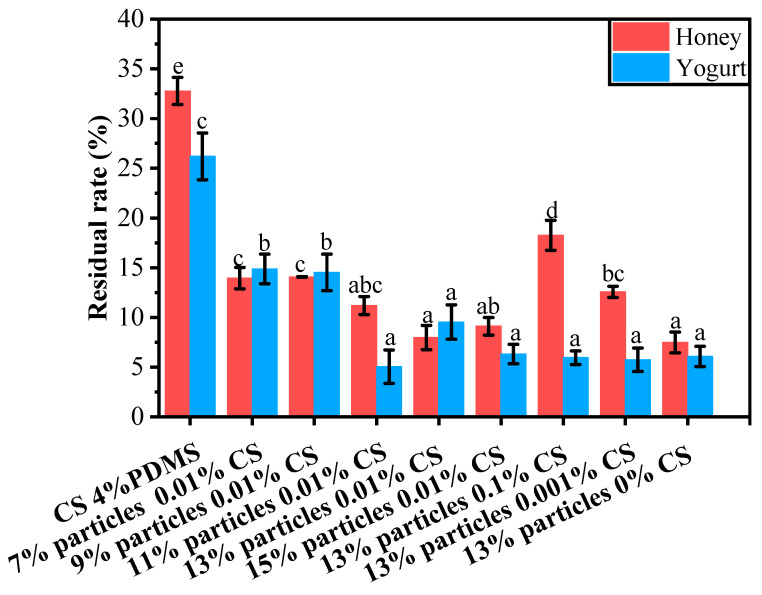
Residual rates of different liquid foods on the surface of the coating films. Different lowercase letters indicate significant differences among samples.

The residual rates of yogurt and honey on the coating films’ surfaces are presented in Figure 11. For honey, the CS 4% PDMS film exhibited a notably higher residue rate compared to the other coating films. This can be attributed to the lack of surface structure in CS 4% PDMS, which allowed for direct contact between the film and honey, thereby increasing the residue rate. 

When the wax particle concentration was below 13%, the residue rate decreased as the concentration of wax particles increased. At a particle concentration of 13%, the residue rate reached its lowest point at 7.97%. This reduction was due to the presence of wax particle protrusions on the coating film’s surface, which enhanced the film’s roughness and decreased the surface interaction between the honey and the film’s surface. However, when the particle concentration became too high, some particles could not be fully covered by PDMS (as indicated by FTIR spectroscopy), leading to an increase in the average residue rate of honey.

As the concentration of CS decreased, the residual rate declined. This is because a lower concentration of CS facilitated the exposure of wax particles, effectively increasing the roughness of the films and minimizing the surface interaction between the liquid food and the films.The trend in the residual rate of yogurt mirrored that of honey; however, the CS concentration did not significantly impact the residual rate of yogurt.

## 4. Conclusions

In this work, TiO_2_-stabilized carnauba wax particles were prepared using the Pickering emulsion method. The resulting carnauba wax particles exhibited a spherical morphology and a hierarchical structure, characterized by nano-scale and micro-scale TiO_2_ aggregates on their surfaces. The size, concentration, and type of TiO_2_ particles significantly influenced the size and number of TiO_2_ aggregates and the sliding angle, while only slightly impacting the contact angle of the wax particles. Hydrophilic TiO_2_ particles measuring 5–10 nm presented smaller sliding angles and particle sizes compared to other groups. The wax particles were prominently visible on the coating films’ surfaces and significantly enhanced the hydrophobicity of the films in comparison to the control. As the concentration of wax particles increased and the concentration of CS decreased, the hydrophobicity of the coating films improved, resulting in a reduced residue rate of liquid foods on the films. Mechanical durability tests indicated that the coating films provided a certain level of protection for the wax particles.

This study demonstrates that the Pickering emulsion method is an effective approach for preparing hierarchical wax particles. Furthermore, blending these wax particles with a diluted chitosan solution can successfully maintain their structural integrity, thereby enhancing the hydrophobic characteristics of chitosan-based materials.

## Data Availability

The data generated for this study are available upon request.
